# The Common Diseases of Children

**Published:** 1884-01

**Authors:** 


### The Common Diseases of Children.

Dr. R. L. Moore read a paper on this subject before the last meeting of the American Medical Association (*Ass. Journal*, October 6, 1883), which concludes as follows:

One of the watchwords in treating children in "elimination." Don't lock up the secretions. Give Nature, that grand old mother, a chance. Very rarely should opium, or any of its preparations or derivatives, be used in the treatment of children. He who abides the nearest to this rule will always have the best succss in treating them. Look after them closely. Stand by the small and frequently-repeated doses of tasteless medicines. Never forget that a sick child is always dangerously sick.

